# Marine Algal Metabolites as Cellular Antioxidants: A Study of Caulerpin and Caulerpinic Acid in *Saccharomyces cerevisiae*

**DOI:** 10.3390/md23090338

**Published:** 2025-08-25

**Authors:** Graziana Assalve, Paola Lunetti, Annalisa Fai, Antonio Terlizzi, Vincenzo Zara, Alessandra Ferramosca

**Affiliations:** 1 Department of Experimental Medicine, University of Salento, 73100 Lecce, Italy; graziana.assalve@unisalento.it (G.A.); paola.lunetti@unisalento.it (P.L.); annalisa.fai@unisalento.it (A.F.); vincenzo.zara@unisalento.it (V.Z.); 2 Department of Life Sciences, University of Trieste, 34127 Trieste, Italy; antonio.terlizzi@szn.it; 3 Stazione Zoologica Anton Dohrn, Villa Comunale, 80121 Napoli, Italy

**Keywords:** algal metabolites, *Caulerpa*, antioxidants, oxidative stress, *S. cerevisiae*

## Abstract

Oxidative stress caused by excessive reactive oxygen species (ROS) contributes to numerous chronic diseases. Marine green algae of the *Caulerpa* genus are rich in bioactive compounds with potential antioxidant activity. Objective: This study aimed to evaluate the intracellular antioxidant effects of caulerpin (CAU) and its derivative caulerpinic acid (CA) using *Saccharomyces cerevisiae* as a eukaryotic model. Methods: Yeast cells were pretreated with 1 μM of CAU or CA, or with 1 μM of resveratrol (RESV) as a positive control, then exposed to 2 mM of H_2_O_2_. Growth, ROS levels, oxidative damage markers, and antioxidant defenses were assessed. Results: Both CAU and CA significantly improved cell survival under oxidative stress, restoring growth rates (CAU: 0.129 h^−1^, CA: 0.137 h^−1^) and doubling times (CAU: 5.38 h, CA: 5.07 h) close to control values. Intracellular ROS accumulation, protein carbonylation, and lipid peroxidation were reduced to near-baseline levels. While catalase (Cat) and superoxide dismutase (Sod) activity remained unchanged, CAU and CA elevated intracellular glutathione (GSH) levels (1.6–1.8 fold) and preserved glutathione peroxidase (GPx) activity, compared to stressed cells without antioxidant pretreatment. Conclusions: CAU and CA act as effective intracellular antioxidants, primarily via ROS scavenging and GSH-dependent pathways. These findings support their potential as natural candidates for developing antioxidant-based therapies against ROS-related disorders.

## 1. Introduction

In recent years, marine macroalgae have garnered growing attention as promising sources of novel bioactive compounds with significant therapeutic potential. Among these, algal metabolites are of particular interest due to their remarkable structural diversity and biological specificity. Within this context, green algae of the genus *Caulerpa* have emerged as noteworthy candidates owing to their rich and varied chemical composition.

These tropical marine algae, commonly known as “sea grapes,” are widely consumed across Southeast Asia and the Indo-Pacific, both as nutritious foods and as components of traditional medicine. In addition to their favorable nutritional profiles, characterized by high levels of carbohydrates, proteins, dietary fibers, and essential amino acids [1,2], *Caulerpa* species are prolific producers of a diverse range of bioactive compounds, including phenolic acids, flavonoids, sterols, and indole alkaloids. These metabolites are increasingly being investigated for their potent antioxidant, anti-inflammatory, antidiabetic, and anticancer properties [3,4,5,6,7,8].

Members of the *Caulerpa* genus are predominantly found in shallow, warm-water marine environments, particularly in tropical and subtropical regions. They thrive in sandy or muddy substrates, seagrass beds, coral reefs, and rocky intertidal zones, often forming dense mats or meadows. Some species are known for their invasive potential and ecological impact in non-native habitats, which makes their biological and ecological study particularly relevant [9].

Among the various biological activities exhibited by *Caulerpa*-derived metabolites, their antioxidant potential is especially significant in the context of oxidative stress. This condition occurs when the production of reactive oxygen species (ROS), highly reactive molecules containing oxygen, exceeds the cell’s ability to neutralize them through antioxidant defense systems. Under normal conditions, ROS play essential roles in cell signaling and homeostasis; however, when produced in excess, they can cause oxidative damage to critical biomolecules such as DNA, proteins, and lipids [10]. This redox imbalance is a key factor in the onset and progression of various chronic diseases, including metabolic disorders, cancer, cardiovascular and neurological conditions, infertility, and aging [11,12,13,14,15,16,17].

Marine algae-derived antioxidants have shown considerable promise in mitigating oxidative damage at the cellular level, thus contributing to disease prevention and therapeutic intervention [9,18]. The antioxidant properties of *Caulerpa* species have been demonstrated in various extracts, with dose-dependent activity. These effects are strongly correlated with the alga’s content of total phenolics and flavonoids, particularly in polar and semi-polar solvent fractions [3].

**Figure 1:**
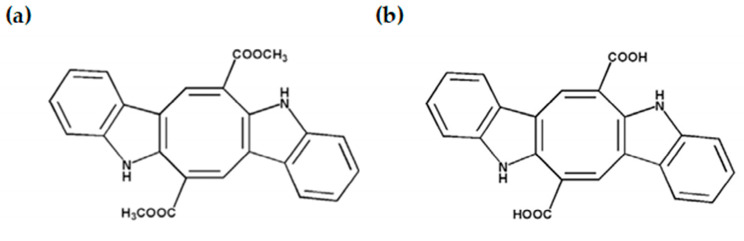
(**a**) Caulerpin (CAU) and (**b**) caulerpinic acid (CA) structures.

Among the most abundant metabolites in *Caulerpa*, caulerpin (CAU) stands out as a bisindole alkaloid characterized by a planar and highly conjugated structure, featuring two indole rings and two methyl ester groups (Figure 1a). This unique structure underlies its wide range of bioactivities, positioning CAU as a lead compound in drug discovery efforts. CAU has been reported to possess antitumor, antimicrobial, and anti-inflammatory properties, as well as inhibitory effects on enzymes involved in metabolic diseases [8,19,20]. *In silico* docking studies have revealed that CAU possesses strong binding affinity for key molecular targets such as α-glucosidase and estrogen receptor alpha, supporting its therapeutic relevance in both diabetes and hormone-related cancers [5,21].

Interestingly, caulerpinic acid (CA) is a structurally related derivative of CAU that naturally co-occurs in *Caulerpa* species (Figure 1b). While CA shares the bisindolic backbone of CAU, it differs by possessing free carboxylic acid groups in place of methyl esters, a variation that may confer distinct physicochemical properties and biological activities. Despite being less well characterized, CA is believed to result from the metabolic transformation or degradation of CAU and may exert complementary or even unique biological effects. However, its antioxidant potential remains largely unexplored.

Supporting the bioactive potential of both compounds, *in vitro* studies using extracts from *C. cylindracea* (a source of CAU and CA) have demonstrated antimicrobial and antiproliferative activity against various cancer cell lines, including colorectal and breast models [22,23,24]. Notably, the selectivity index of crude polyphenolic extracts was markedly higher than that of conventional anticancer drugs, indicating preferential cytotoxicity toward malignant cells while sparing normal ones. These properties make *C. cylindracea* and its metabolites particularly attractive in the search for multifunctional agents capable of targeting multiple hallmarks of disease [23,24].

Despite promising evidence, the intracellular antioxidant effects of CAU and, especially, CA have not been systematically evaluated in eukaryotic models that enable detailed insights into redox regulation and stress response mechanisms. Yeast (*Saccharomyces cerevisiae*), with its conserved eukaryotic pathways and genetic tractability, offers an ideal model for studying the cellular dynamics of oxidative stress and evaluating the biological activity of natural compounds under controlled conditions [25].

The present study investigates the antioxidant potential of CAU and CA in *S. cerevisiae*, with the aim of elucidating their mechanisms of action. By integrating functional assays with molecular markers of oxidative damage and defense, we assess the extent to which each compound can modulate redox homeostasis. In doing so, we provide new insight into the intracellular activity of these *Caulerpa*-derived metabolites and their potential as lead candidates for the development of natural antioxidant therapies. To our knowledge, this is the first study to investigate the *in vivo* antioxidant activity of CAU and CA in *S. cerevisiae*, providing novel insight into their cellular effects and contribution to redox homeostasis.

## 2. Results

### 2.1. Effects of Marine-Derived Compounds on Yeast Cell Viability

**Figure 2:**
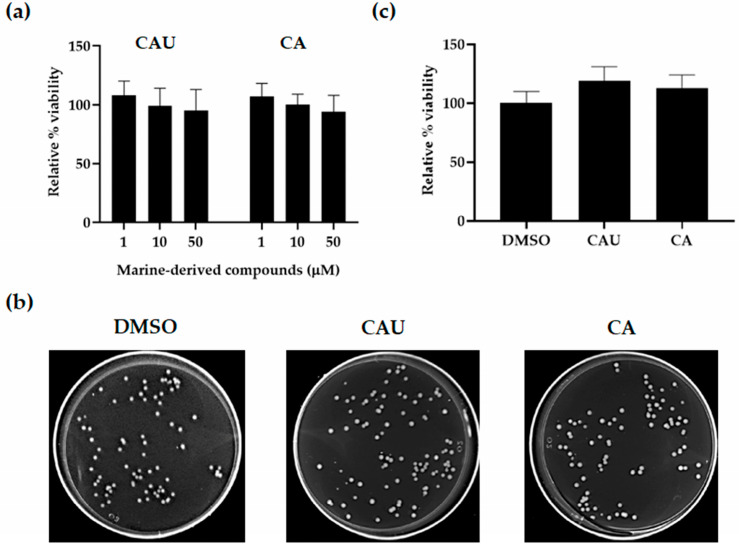
Effects of marine-derived compounds on *S. cerevisiae* cell viability. (**a**) Yeast cells were cultured in synthetic complete (SC) medium supplemented with 2% glucose and treated with increasing concentrations of CAU and CA. Cell growth was assessed after 24 h by measuring optical density at 600 nm (OD_600_) and expressed as relative growth (%) normalized to untreated cells in DMSO. (**b**) For colony-forming units (CFU) analysis, exponentially growing cells were plated on glucose-supplemented SC plates containing 1 µM of each compound. Representative images were taken 24 h after cell seeding. (**c**) Quantification of CFU expressed as relative viability (%), with the DMSO-treated control set at 100%. Data represent the mean ± SD of three independent experiments.

To assess yeast viability, *S. cerevisiae* cells were treated with increasing concentrations (1–50 µM) of CAU and CA, and growth was evaluated by optical density at 600 nm (OD_600_) measurements and colony-forming unit (CFU) counts relative to DMSO controls (Figure 2).

No cytotoxic effects were observed for either compound within the tested concentration range (1–50 µM), as yeast cells exhibited approximately 100% growth relative to the untreated control. Since cell proliferation was not affected, subsequent experiments aimed at evaluating antioxidant activity were conducted using the lowest concentration (1 µM) that resulted in maximal growth comparable to that of the control.

### 2.2. Antioxidant Properties of Marine-Derived Compounds

**Figure 3:**
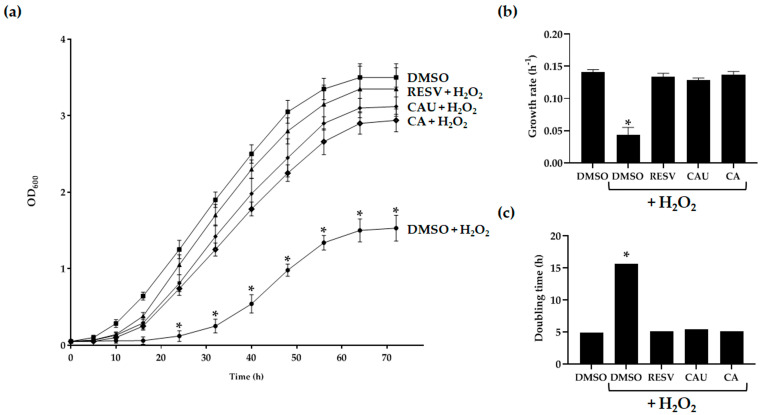
Effects of marine-derived compounds on *S. cerevisiae* growth under oxidative stress conditions. (**a**) Yeast cells were cultured in glucose-supplemented SC medium containing 2 mM of H_2_O_2_, either in the presence or absence of 1 μM of CAU, CA, or resveratrol (RESV). As a control, cells were grown in the absence of H_2_O_2_ and treated with DMSO alone. OD_600_ was measured at the indicated time points to monitor cell growth. Data represent the mean ± SD of three independent experiments. (**b**) Growth rates and (**c**) doubling times were calculated from the growth curves shown in (**a**). Growth rate data are the mean ± SD from three biological replicates, each performed in triplicate. * *p* < 0.005.

Yeast cells grown under fermentative conditions showed reduced viability upon exposure to 2 mM of H_2_O_2_ (Figure 3a).

Treatment with 1 µM of CAU, CA, or resveratrol (RESV), a well-characterized natural antioxidant used as a positive control [26], significantly improved cellular tolerance to oxidative stress compared to cells treated with H_2_O_2_ alone (Figure 3a). Growth kinetics analysis revealed that both CAU- and CA-treated cells exhibited significantly enhanced proliferation, as evidenced by increased growth rates and reduced doubling times relative to the negative control. These effects were comparable to those observed in RESV-treated cultures (Figure 3b,c; Table 1).

To comprehensively evaluate the antioxidant potential of CAU and CA, a series of complementary growth recovery assays were performed under oxidative stress conditions induced by 2 mM of H_2_O_2_, including spot assays, drug drop tests, and CFU assays.

**Figure 4:**
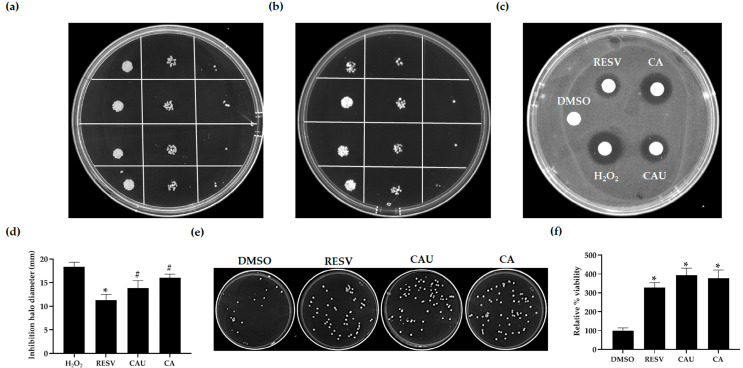
Evaluation of the antioxidant effects of marine-derived compounds in *S. cerevisiae* under H_2_O_2_-induced stress. (**a**) Yeast cells were grown to the exponential phase in SC medium supplemented with glucose and treated with either 1 μM of the indicated compounds or DMSO (control). Cultures were serially diluted and spotted onto control SC plates and (**b**) SC plates containing 2 mM H_2_O_2_. Images were acquired after 48 h of incubation at 30 °C and are representative of three independent replicates. (**c**) Approximately 5.5 × 10^5^ cells were spread onto a glucose-supplemented SC agar plate. Sterile filter disks were placed on the surface and loaded with 5 μL of the tested compounds (1 μM) combined with 5 μL of 2 mM H_2_O_2_. Two control disks were loaded with either DMSO alone or H_2_O_2_ alone. (**d**) After 72 h of incubation at 30 °C, the diameters of the inhibition halos around the filters were measured to evaluate cell sensitivity to oxidative stress, with statistical significance determined relative to the halo produced by H_2_O_2_ alone. (**e**) Cultures pretreated for 24 h with either the compounds (1 μM) or DMSO were plated on SC agar containing 2 mM of H_2_O_2_ to perform CFU assays. Images were taken after 48 h of incubation at 30 °C and are representative of three independent experiments. (**f**) Viability was quantified as the percentage of colonies relative to the DMSO control (set as 100%). Data are shown as mean ± SD from three independent experiments. # *p* < 0.05; * *p* < 0.005.

In spot assays, yeast cultures pretreated with either CAU or CA exhibited markedly improved survival on glucose-supplemented SC plates containing H_2_O_2_ compared to the DMSO-treated control, indicating enhanced oxidative stress resistance. In the absence of H_2_O_2_, no differences in growth were observed among the treated and control groups, confirming that the compounds did not interfere with cell proliferation (Figure 4a,b).

To further validate these findings, the drug drop test was employed. Filter disks impregnated with H_2_O_2_ alone or in combination with each compound were placed on SC-glucose plate seeded with yeast cells. A notable reduction in the inhibition halo was observed around filters containing H_2_O_2_ plus CAU or CA compared to H_2_O_2_ alone, supporting the compounds’ capacity to mitigate oxidative stress (Figure 4c,d).

Consistent results were obtained in CFU assays. Yeast cultures treated with CAU or CA and then plated on SC-glucose agar with H_2_O_2_ formed significantly more colonies than those treated with DMSO, indicating improved cell survival and reproductive capacity under stress conditions (Figure 4e,f).

In all assays, CAU and CA displayed a protective effect comparable to that of RESV. Taken together, these findings indicate that both compounds confer substantial protection against H_2_O_2_-induced cytotoxicity in yeast, enhancing cellular survival and promoting growth under oxidative stress conditions, likely through antioxidant mechanisms.

### 2.3. Reduction of Intracellular ROS and Oxidative Damage by Marine-Derived Compounds

**Figure 5:**
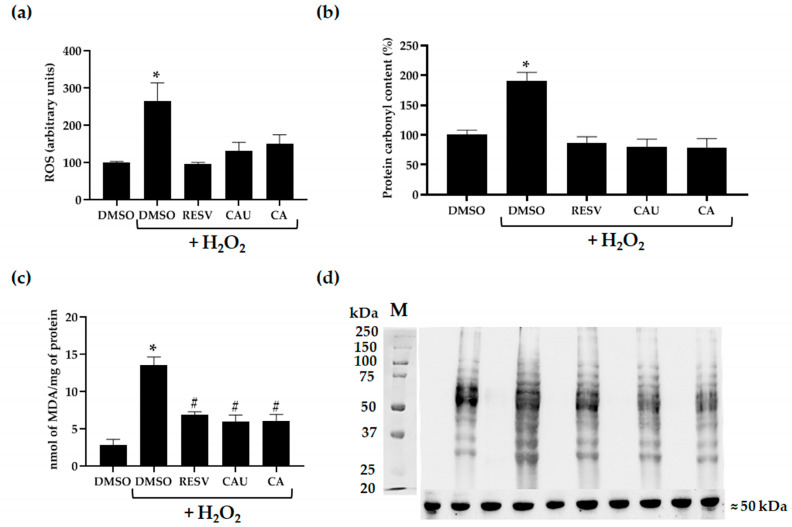
Evaluation of intracellular oxidative stress and damage markers in yeast cells. (**a**) Intracellular ROS levels were measured using the 2′,7′-dichlorofluorescein diacetate (H_2_DCF-DA) fluorescent probe in yeast cells pretreated with DMSO, CAU, CA, or RESV and subsequently exposed to 2 mM of H_2_O_2_. Data represent the mean ± SD from at least three independent experiments, each performed in triplicate. (**b**) Protein carbonylation was assessed as a marker of oxidative protein damage in cells pretreated with the indicated compounds and then treated with H_2_O_2_. Quantification was performed by densitometric analysis of the immunoblot shown in (**d**). Protein carbonyl levels in DMSO-treated control cells were set to 100%. (**c**) Lipid peroxidation was evaluated using the thiobarbituric acid-reactive substances (TBARS) assay to measure malondialdehyde (MDA) levels in cultures pretreated with DMSO or the indicated compounds and then incubated with or without H_2_O_2_. Results are presented as mean ± SD (*n* = 3). (**d**) Representative immunoblot of protein carbonyl content, with α-tubulin used as loading control. # *p* < 0.05; * *p* < 0.005.

To explore the antioxidant mechanism of CAU and CA, intracellular ROS levels were measured using the H_2_DCF-DA probe [27]. Yeast cultures were pretreated with DMSO, CAU, or CA, and subsequently exposed to 2 mM of H_2_O_2_. As expected, DMSO-pretreated cells exhibited a marked increase in intracellular ROS following oxidative stress. In contrast, cells pretreated with CAU or CA showed a significantly reduced ROS accumulation, comparable to that observed in unstressed cells or those pretreated with the known antioxidant RESV (Figure 5a). These results indicate that both compounds effectively scavenge ROS at the tested concentration *in vivo*.

Given their apparent scavenging activity, we next assessed whether CAU and CA could also mitigate the cellular damage typically induced by oxidative stress. Exposure to H_2_O_2_ is known to trigger the formation of multiple ROS species, including hydroxyl radicals (•OH) and singlet oxygen (^1^O_2_), which can damage vital macromolecules such as lipids and proteins [28]. To evaluate oxidative damage, we measured levels of protein carbonylation and lipid peroxidation, both established biomarkers of ROS-induced cellular injury.

Consistent with elevated oxidative stress, H_2_O_2_-treated cultures displayed a significant increase in both protein carbonyl content and malondialdehyde (MDA), a byproduct of lipid peroxidation, compared to the DMSO control (Figure 5b–d). However, pretreatment with CAU or CA substantially reduced protein carbonylation, restoring levels to those observed in unstressed control cultures and in cells treated with RESV (Figure 5b,d). Lipid peroxidation was also significantly reduced in cells pretreated with CAU or CA compared to those pretreated with DMSO prior to H_2_O_2_ exposure, although levels did not return completely to baseline (Figure 5c).

Overall, these findings demonstrate that CAU and CA provide effective protection against H_2_O_2_-induced oxidative damage in yeast cells. Their ability to lower intracellular ROS and reduce oxidative damage to proteins and lipids suggests a direct ROS scavenging mechanism underlying their antioxidant effects.

### 2.4. Effect of Marine-Derived Compounds on the Enzymatic and Non-Enzymatic Defense Systems

**Figure 6:**
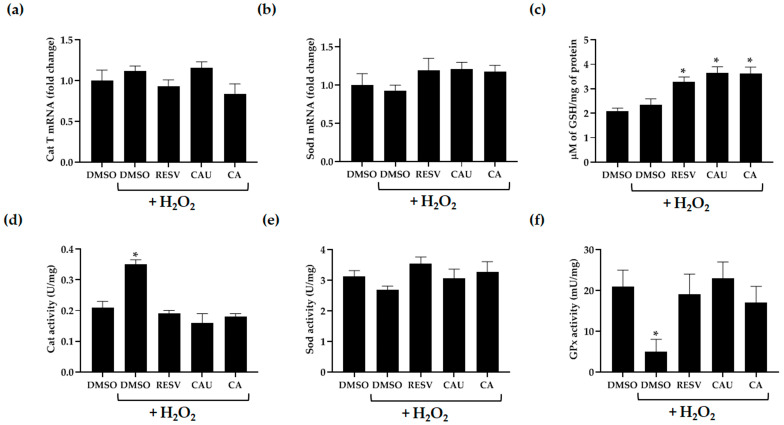
Measurement of antioxidant enzymatic and non-enzymatic defense systems. (**a**) Relative mRNA expression levels of catalase T (Cat T) and (**b**) superoxide dismutase 1 (Sod1) were measured in yeast cells pretreated for 2 h with the indicated compounds (1 µM), followed by exposure to 2 mM of H_2_O_2_ for 1 h. (**c**) Intracellular levels of reduced glutathione (GSH), (**d**) Cat, (**e**) Sod, and (**f**) glutathione peroxidase (GPx) activity were assessed under the same treatment conditions. All data represent the mean ± SD of three independent biological replicates. * *p* < 0.005.

To investigate whether the antioxidant effects of CAU and CA involve endogenous antioxidant defenses, we analyzed the expression levels and enzymatic activities of catalase (Cat) and superoxide dismutase (Sod) in yeast under oxidative stress conditions (Figure 6a,b,d,e). These enzymes represent key components of the yeast enzymatic antioxidant system, with isoforms localized in different subcellular compartments and known to respond variably to oxidative stress [29,30,31,32,33].

Exposure to 2 mM of H_2_O_2_ did not significantly affect the expression levels of Cat T and Sod1, regardless of compound pretreatment (Figure 6a,b). However, H_2_O_2_ treatment resulted in a significant increase in Cat enzymatic activity, which returned to basal levels in cells that were pretreated with the tested compounds (Figure 6d). This restoration is likely due to the compounds’ ROS scavenging ability, which may have reduced intracellular H_2_O_2_ levels, thereby decreasing the demand for elevated Cat activity. In contrast, no appreciable changes in Sod activity were observed across any of the experimental conditions, including untreated, stressed, and compound-pretreated stressed cells (Figure 6e).

To evaluate the effects of the marine-derived compounds on non-enzymatic antioxidant systems [34,35,36], intracellular reduced glutathione (GSH) levels and glutathione peroxidase (GPx) enzymatic activity were assessed in yeast cells pretreated with CAU or CA and subsequently exposed to H_2_O_2_ (Figure 6c,f). Compared to DMSO-treated control cells, both CAU- and CA-pretreated cells showed a marked increase in GSH levels, ranging from 1.6- to 1.8-fold, similar to the increase observed in cells pretreated with the reference antioxidant RESV. Notably, no significant increase in GSH was detected in DMSO-pretreated cells following H_2_O_2_ exposure, confirming that the elevation in GSH levels was specifically associated with the antioxidant pretreatments (Figure 6c).

In parallel, H_2_O_2_ exposure resulted in a substantial decrease in GPx activity in untreated cells, indicating oxidative stress-induced enzyme impairment. However, pretreatment with CAU or CA effectively preserved GPx activity, reaching levels nearly four times higher than those observed in H_2_O_2_-stressed cells without antioxidant pretreatment. This protective effect was comparable to that of RESV (Figure 6f), suggesting that the tested compounds mitigate oxidative stress not only by directly scavenging ROS but also by preserving the activity of key redox enzymes.

Together, these findings indicate that CAU and CA enhance the intracellular antioxidant capacity by sustaining GSH levels and preventing the inactivation of GPx under oxidative stress. This dual effect likely contributes to the improved cell survival and reduced ROS accumulation observed in previous assays, underscoring the critical role of non-enzymatic antioxidant pathways in the protective action of these marine-derived compounds.

## 3. Discussion

Oxidative stress, caused by excessive accumulation of ROS from both endogenous and exogenous sources, is a well-established contributor to cellular damage and plays a central role in the development of many chronic diseases such as cancer, cardiovascular and neurodegenerative disorders, diabetes, and aging [10,17,37,38,39]. Due to its fundamental involvement in these multifactorial conditions, natural antioxidants are increasingly studied as preventive agents, particularly in disorders characterized by redox imbalance.

Marine algae are a valuable source of bioactive compounds, including phenolic acids, flavonoids, sterols, and indole alkaloids, which exhibit antioxidant, anti-inflammatory, antitumor, and metabolic regulatory effects [3,4,5,6,7,8,40]. Among these, *C. cylindracea* is distinguished by its high content of phenolics and flavonoids, which contribute to its potent antioxidant capacity [3]. One of its principal metabolites, CAU, a bisindole alkaloid, has demonstrated antitumor, antimicrobial, and anti-inflammatory activities, along with modulation of metabolic enzymes [8,19,20,22,23,24].

This study utilized the eukaryotic model organism *S. cerevisiae* to explore the intracellular antioxidant potential of CAU and its derivative CA under oxidative stress induced by H_2_O_2_. Yeast offers a highly conserved redox regulatory network comparable to higher eukaryotes and allows for tightly controlled experimental conditions [25]. Using this approach, we examined the protective effects of CAU and CA in yeast cells exposed to H_2_O_2_-induced oxidative stress. The effects of CAU and CA were benchmarked against RESV, a well-known antioxidant polyphenol.

Initial toxicity assessments confirmed that CAU and CA did not adversely affect yeast viability within the tested concentration range, allowing further evaluation of their antioxidant effects. When exposed to sublethal H_2_O_2_ concentrations, yeast cultures supplemented with CAU or CA showed significantly reduced growth impairment, as observed with RESV-treated culture, compared to culture without pretreatment. These results indicate that CAU and CA effectively enhance cell survival under oxidative challenge.

The protective mechanism appears to involve direct ROS scavenging, likely related to the aromatic bisindole structure of these compounds. Intracellular ROS measurements revealed that pretreatment with CAU or CA maintained ROS at basal levels despite H_2_O_2_ exposure, whereas untreated cells accumulated significant ROS. Correspondingly, markers of oxidative damage (protein carbonylation and lipid peroxidation) were markedly reduced in pretreated cells, further supporting a role in mitigating oxidative injury.

Beyond direct ROS neutralization, CAU and CA may modulate endogenous antioxidant defenses. In *S. cerevisiae*, enzymatic antioxidants such as Sod, Cat, and peroxidases, alongside the non-enzymatic GSH system, are essential for redox homeostasis [41].

Previous studies have shown that exposure to H_2_O_2_ can elicit either an upregulation or suppression of antioxidant enzyme expression and activity, depending on the concentration of the oxidant and the specific characteristics of the yeast strain used [29]. Additionally, during exponential growth, Sod1 and Cat T expression may remain unaltered even following oxidative challenge [42]. Our data align with prior studies showing that moderate H_2_O_2_ stress does not significantly alter Sod1 or Cat T expression or Sod enzymatic activity [29,30]. Notably, Cat activity increased only in H_2_O_2_-treated cells without compound pretreatment, suggesting that the antioxidant compounds prevent the need for upregulated Cat by reducing intracellular H_2_O_2_.

In contrast, intracellular GSH levels increased significantly in cultures pretreated with CAU, CA, or RESV compared to stressed cells without pretreatment, which maintained basal GSH levels. This suggests an induced enhancement of non-enzymatic antioxidant capacity by these compounds. Since GSH serves as a reducing cofactor for GPx, this may reflect a strategic shift in antioxidant defenses from Cat-dependent to GPx-mediated detoxification [36].

GPx activity, known to be impaired by oxidative stress, possibly via post-translational modifications rather than GSH depletion, was preserved in pretreated cells at levels comparable to unstressed controls. This preservation likely contributes significantly to the improved cellular viability observed, as GPx activity is critical for survival during oxidative insult [43].

In summary, CAU and CA exhibit cytoprotective effects through a dual mechanism: direct ROS scavenging via their bisindole structure and enhancement of non-enzymatic redox buffering through increased GSH levels and maintenance of GPx activity.

These findings provide a mechanistic framework supporting the potential application of these marine-derived alkaloids as natural antioxidants to counteract oxidative stress-related cellular damage.

## 4. Materials and Methods

### 4.1. Purification and Alkaline Hydrolysis of CAU

*C. cylindracea* was collected by scuba divers off the southern coast of Italy and extracted with acetone at room temperature as detailed in [8,44]. After solvent evaporation under reduced pressure, the extract was purified through diethyl ether extraction, thin-layer chromatography (TLC), and Sephadex LH-20 chromatography. Pure CAU was identified by ^1^H and ^13^C NMR. CAU was then hydrolyzed and purified to obtain CA, confirmed by ^1^H NMR [8].

### 4.2. Yeast Strains and Growth Conditions

The wild-type strain of *S. cerevisiae* BY4742 (MATα, *his*3Δ1, *leu*2Δ0, *lys*2Δ0, *ura*3Δ0) was obtained from the EUROFAN resource center EUROSCARF (Frankfurt, Germany).

Yeast cells were grown at 30 °C either in YP medium (2% [*w*/*v*] bactopeptone, CAS: 73049-73-7; 1% [*w*/*v*] yeast extract, CAS: 8013-01-2; pH 4.8) or in SC medium (0.67% [*w*/*v*] yeast nitrogen base, 0.1% [*w/v*] drop-out mix, pH 4.5). All culture media components were purchased from Becton, Dickinson and Company (Franklin Lakes, NJ, USA).

Cells were pre-cultured overnight in YP medium supplemented with 2% (*w*/*v*) glucose (CAS: 50-99-7) at 120 rpm, using an orbital shaker incubator at 30 °C. Cells were then harvested, washed, and resuspended in SC medium supplemented with 2% (*w*/*v*) glucose, adjusting the final OD_600_ to 0.1. Separate cultures were set up for each treatment, incubated until reaching the exponential phase, and subsequently used for all experiments. For solid media, 2% (*w*/*v*) agar (CAS: 9002-18-0) was added [45].

### 4.3. Evaluation of S. cerevisiae Cells’ Sensitivity to Marine-Derived Compounds

Exponentially growing wild-type cells were treated with varying concentrations (1–50 μM) of CAU and CA in SC liquid medium supplemented with glucose. After 24 h of incubation, cell growth was assessed by measuring OD_600_. Relative growth (%) was calculated by normalizing the OD_600_ values to those of untreated control cells incubated with DMSO, the solvent used to dissolve the compounds.

CFU assays were performed by spreading appropriate cell dilutions onto glucose-supplemented SC agar plates containing 1–50 μM of each compound. After 24 h of incubation, colonies were counted and cell viability was expressed as a percentage relative to the DMSO (CAS: 67-68-5) control, which was set at 100%.

### 4.4. Cellular Assays for the Evaluation of Antioxidant Activity

Exponentially growing wild-type cells were treated with 1 μM of CAU or CA for 2 h, followed by exposure to 2 mM of H_2_O_2_ (CAS: 7722-84-1). Growth curves were generated by measuring OD_600_ at regular intervals, and growth parameters such as doubling time and growth rate were calculated [46].

For spot assays, cells were pretreated with the compounds for 2 h, serially diluted, and spotted onto glucose-supplemented SC agar plates with or without 2 mM H_2_O_2_. Plates were incubated at 30 °C for 2 days.

For CFU assays, cells were treated as described above and plated in triplicate onto SC agar plates supplemented with 2 mM of H_2_O_2_. After 2 days of incubation at 30 °C, colonies were counted, and viability was expressed as a percentage relative to the untreated control (set at 100%).

For the drug drop test, cells were spread onto SC agar plates supplemented with glucose. Sterile filters were placed on the agar surface and spotted with the test compounds. After 1 h, 2 mM of H_2_O_2_ was applied to the same filters. Negative and positive controls were included by spotting 2 mM H_2_O_2_ alone or DMSO alone, respectively. Plates were incubated for 3 days at 30 °C, after which the diameter of the inhibition halo around each disk was measured [47].

In all experiments, RESV-treated cultures were included as a positive control.

### 4.5. Estimation of Intracellular Oxidation

Exponentially growing wild-type cells were either directly exposed to 2 mM H_2_O_2_ or pretreated with the tested compounds (1 µM) for 2 h at 30 °C in a shaker incubator. After treatment, cells were collected by centrifugation at 3000× *g* for 5 min and used for intracellular ROS quantification.

ROS levels were assessed using H_2_DCF-DA (CAS: 127770-45-0), a non-fluorescent probe that was hydrolyzed and oxidized within the cell to form the highly fluorescent compound 2′,7′-dichlorofluorescein (DCF). Cells were washed with sterile ddH_2_O and incubated with 20 µM H_2_DCF-DA in the dark for 30 min at 30 °C with shaking at 150 rpm. After incubation, cells were centrifuged at 3000× *g* for 5 min and washed twice with sterile ddH_2_O. A final centrifugation step was performed under the same conditions, and the resulting supernatant was transferred to a black 96-well microplate for fluorescence measurement. Fluorescence was recorded using a microplate reader with excitation/emission wavelengths of 485/535 nm [34].

### 4.6. Preparation of Yeast Cell–Free Extracts

For the determination of protein carbonyl content, lipid peroxidation, GSH levels, and enzymatic activities, cell-free extracts were prepared from yeast cultures grown to the exponential phase. Cultures were pretreated with the tested compounds (1 µM) for 2 h, followed by exposure to 2 mM of H_2_O_2_ for 1 h. After treatment, cells were harvested by centrifugation at 2000× *g* for 5 min and washed twice with potassium phosphate buffer supplemented with 1 mM phenylmethylsulfonyl fluoride (PMSF, CAS: 329-98-6). Cell lysis was carried out by resuspending the pellet in lysis buffer (50 mM Tris–HCl, CAS: 77-86-1; 150 mM NaCl, CAS: 7647-14-5; 50 mM EDTA, CAS: 6381-92-6; 1 mM PMSF, pH 7.2) and adding an equal volume of acid-washed glass beads (425–600 µm). Cell disruption was performed by alternating 1 min vortexing with 1 min cooling on ice, repeated 8 times. Lysates were centrifuged at 14,000× *g* for 15 min at 4 °C to remove cell debris, and the resulting supernatants were collected as cell-free extracts. Protein concentration was determined using the Bradford method, with bovine serum albumin (BSA, CAS: 9048-46-8) as the standard [36].

### 4.7. Measurement of Oxidative Damage

Protein carbonyl content was detected in the cell-free extracts by using the OxyBlot^TM^ Protein Oxidation Detection Kit (Merck Millipore, Billerica, MA, USA), according to the manufacturer’s instructions. Image analysis was carried out using the ChemiDoc imaging system and Image Lab 6.1.0 software (Bio-Rad Laboratories, Hercules, CA, USA) [48].

Lipid peroxidation was quantified by measuring thiobarbituric acid-reactive substances (TBARS), following the instructions of the Lipid Peroxidation (MDA) Assay Kit (Sigma-Aldrich, St. Louis, MO, USA). Briefly, 200 µL of cell-free extract were mixed with 600 µL of thiobarbituric acid (TBA) reagent (30% glacial acetic acid, CAS: 64-19-7; 1% TBA, CAS: 504-17-6). The addition of the reagent stopped lipid peroxidation and initiated the reaction. Samples were heated in a thermoblock at 95 °C for 1 h, then rapidly cooled on ice. Fluorescence was measured at an excitation/emission wavelength of 530/560 nm. Results were expressed as nanomoles of TBARS per milligram of protein.

### 4.8. Determination of Cat, Sod, and GPx Activity

Cat activity was measured in cell-free extracts using the Catalase Assay Kit (Sigma-Aldrich, St. Louis, MO, USA), following the manufacturer’s protocol. Briefly, 10 µL of extract was incubated with 45 µM of H_2_O_2_ for 30 min at room temperature. Subsequently, a detection reagent containing a dye and horseradish peroxidase (HRP) was added, and fluorescence was measured after 10 min at an excitation/emission wavelength of 530/585 nm. Cat concentration was determined by comparison to a standard curve. One unit of Cat activity is defined as the amount of enzyme that decomposes 1 µmol of H_2_O_2_ per min at pH 7.0 and room temperature.

Sod activity was determined in cell-free extracts using the Superoxide Dismutase Assay Kit (Sigma-Aldrich, St. Louis, MO, USA), following the manufacturer’s instructions. The assay is based on a colorimetric method in which superoxide anions, generated by xanthine oxidase (XO), react with a WST-1 dye to produce a water-soluble formazan dye. Sod competes with the dye for superoxide anions, thereby reducing the formation of the colored product. The absorbance at 440 nm (OD_440_) is inversely proportional to Sod activity and is quantified by comparison with a standard curve.

GPx activity was measured using the Ransel kit (Randox Laboratories, UK), according to the manufacturer’s instructions. The millimolar extinction coefficient of nicotinamide adenine dinucleotide phosphate (NADPH) at 340 nm (6.22 mM^−1^ cm^−1^) was used for calculations of GPx activity, which was expressed as mU per mg protein.

Results are presented as the mean ± SD of experiments from three independent cultures (*n* = 3).

### 4.9. Quantification of Intracellular GSH Levels

GSH content was determined from cell-free extracts. Equal volumes of extract and ice-cold 2 M HClO_4_ (CAS: 7601-90-3) containing 4 mM EDTA were mixed and incubated on ice for 15 min. The mixture was then centrifuged at 2000× *g* for 5 min, and the resulting supernatant was neutralized with 3 M KOH (CAS: 1310-58-3). For the colorimetric assay, 200 µL of the neutralized supernatant was combined with 400 µL of 100 mM phosphate buffer (pH 8.0) and 10 µL of 10 mM DTNB (5,5′-dithiobis-(2-nitrobenzoic acid), CAS: 69-78-3) on ice. After 5 min of incubation, absorbance was measured at 412 nm. GSH levels were expressed as µM of GSH per mg of protein [25]. Data were expressed as the mean ± SD from three independent biological replicates (*n* = 3).

### 4.10. Gene Expression Analysis by Quantitative Real-Time PCR (qRT-PCR)

Yeast cultures were grown to the exponential phase (OD_600_ ≈ 1.5), then pretreated for 2 h with DMSO or one of the compounds of interest (1 μM), followed by exposure to 2 mM of H_2_O_2_ for 1 h. For RNA extraction, approximately 5 × 10^7^ cells per sample were harvested, and total RNA was isolated using the Blood/Tissues Total RNA Extraction Mini Kit (Fisher Molecular Biology, Rome, Italy) according to the manufacturer’s instructions. RNA concentration and purity were assessed using a NanoDrop™ spectrophotometer by measuring A_260/280_ and A_260/230_ ratios, with acceptable values of ~1.8 and 1.8–2.2, respectively.

cDNA was synthesized from 1 μg of total RNA using the PrimeScript^TM^ RT Master Mix (TaKaRa, Kusatsu, Japan). qRT-PCR was performed on a StepOne™ Real-Time PCR System (Applied Biosystems, Invitrogen, Waltham, MA, USA) using TaqMan^®^ chemistry. Each 10 μL reaction mixture contained 1X TaqMan^®^ Gene Expression Master Mix (Applied Biosystems), 0.14 μg of cDNA, nuclease-free water, and 0.9 μM of each TaqMan^®^ Gene Expression Assay primer/probe set specific for CTT1 (Assay ID: Sc04125548_s1), SOD1 (Assay ID: Sc04138965_s1), and TAF10 (Assay ID: Sc04111472_s1), the latter used as the endogenous control. Thermal cycling conditions were as follows: 95 °C for 10 min (initial denaturation), followed by 40 cycles of 95 °C for 15 s and 60 °C for 60 s. All reactions were run in triplicate in three independent biological replicates. Relative gene expression was calculated using the 2^−ΔΔCt^ method, where ΔΔCT indicates [Ct_(target gene)_ − Ct_(TAF10)_]_treated_ − [Ct_(target gene)_ − Ct_(TAF10)_]_control_ [45,49].

## 5. Conclusions

This study demonstrates that the marine-derived metabolites CAU and CA exert potent intracellular antioxidant activity in *S. cerevisiae*. Both compounds effectively counteract oxidative stress by directly scavenging ROS, thereby mitigating macromolecular damage such as protein carbonylation and lipid peroxidation.

Notably, their protective effects are not mediated by the activation of canonical antioxidant enzymes but rather by reinforcing non-enzymatic defenses. Specifically, CAU and CA enhance intracellular GSH levels and preserve GPx activity under oxidative conditions, sustaining redox homeostasis and cell viability.

These findings provide mechanistic insight into the dual antioxidant action of these bisindole alkaloids and underscore their potential as lead compounds for the development of natural therapeutics targeting ROS-related pathologies.

## Figures and Tables

**Table 1:** Growth rate and doubling time of *S. cerevisiae* cultures treated with marine-derived compounds and exposed to oxidative stress. Yeast cells were grown in glucose-supplemented SC medium with or without 2 mM of H_2_O_2_, in the presence of 1 μM of the indicated compounds. Reported values for growth rate are expressed as mean ± SD from three independent experiments, each performed in triplicate.

		Growth Rate (h^−1^)	Doubling Time (h)
−H_2_O_2_	DMSO	0.141 ± 0.004	4.91
+H_2_O_2_	DMSO	0.044 ± 0.011	15.68
	RESV	0.134 ± 0.005	5.16
	CAU	0.129 ± 0.003	5.38
	CA	0.137 ± 0.005	5.07

## Data Availability

The datasets generated and analyzed during this current study are available from the corresponding author upon reasonable request.

## Abbreviations

The following abbreviations are used in this manuscript:

BSA	Bovine Serum Albumin
CA	Caulerpinic Acid
Cat	Catalase
CAU	Caulerpin
CFU	Colony-Forming Units
DCF	2′,7′-dichlorofluorescein
ddH2O	double-distilled water
DMSO	Dimethyl sulfoxide
DTNB	5,5′-dithiobis-(2-nitrobenzoic acid)
GPx	Glutathione Peroxidase
GSH	Glutathione
H_2_DCF-DA	2′,7′-dichlorofluorescein diacetate
HRP	Horseradish peroxidase
MDA	Malondialdehyde
NADPH	Nicotinamide adenine dinucleotide phosphate
OD	Optical Density
PMSF	Phenylmethylsulfonyl fluoride
qRT-PCR	Quantitative Real-Time PCR
RESV	Resveratrol
ROS	Reactive Oxygen Species
SC	Synthetic Complete
SD	Standard Deviation
Sod	Superoxide dismutase
TBA	Thiobarbituric acid
TBARS	Thiobarbituric acid-reactive substances
TLC	Thin-Layer Chromatography
XO	Xanthine Oxidase
