# Correction: Assalve et al. Marine Algal Metabolites as Cellular Antioxidants: A Study of Caulerpin and Caulerpinic Acid in *Saccharomyces cerevisiae*. *Mar. Drugs* 2025, *23*, 338

**DOI:** 10.3390/md24060207

**Published:** 2026-06-11

**Authors:** Graziana Assalve, Paola Lunetti, Annalisa Fai, Antonio Terlizzi, Vincenzo Zara, Alessandra Ferramosca

**Affiliations:** 1 Department of Experimental Medicine, University of Salento, 73100 Lecce, Italy; graziana.assalve@unisalento.it (G.A.); paola.lunetti@unisalento.it (P.L.); annalisa.fai@unisalento.it (A.F.); vincenzo.zara@unisalento.it (V.Z.); 2 Department of Life Sciences, University of Trieste, 34127 Trieste, Italy; antonio.terlizzi@szn.it; 3 Stazione Zoologica Anton Dohrn, Villa Comunale, 80121 Napoli, Italy

## Text Correction

Following the publication of this article [1], the authors would like to include the following Acknowledgments:

“The authors gratefully acknowledge Ernesto Mollo (Institute of Biomolecular Chemistry, National Research Council of Italy, ICB-CNR) for the provision of caulerpin and caulerpinic acid used in this study.”

The authors state that the scientific conclusions are unaffected. This correction was approved by the Academic Editor. The original publication has also been updated.
